# Time Farms Stay Naïve for Porcine Reproductive and Respiratory Syndrome

**DOI:** 10.3390/ani13020310

**Published:** 2023-01-16

**Authors:** Mariana Kikuti, Catalina Picasso-Risso, Claudio Marcello Melini, Cesar A. Corzo

**Affiliations:** Department of Veterinary Population Medicine, University of Minnesota, Saint Paul, MN 55108, USA

**Keywords:** porcine reproductive and respiratory syndrome, epidemiology, prevention and control, disease eradication

## Abstract

**Simple Summary:**

Porcine Reproductive and Respiratory Syndrome (PRRS) is one of the main infectious diseases affecting swine herds in the U.S. Control and elimination are facing challenges, including the absence of a vaccine that confers sterilizing immunity. Elimination through herd closure can also be a long, uncertain, and costly process, with herds often taking around 41 weeks to start consistently weaning virus-free piglets after an outbreak is detected. Thus, producers and practitioners may hesitate in pursuing elimination if there is a perception that the herd will soon face another PRRS outbreak, opting instead to maintain some level of immunity in the herd indefinitely. Of all the breeding herds monitored over 12 years, only about 1/6 eliminated PRRS from their herds and remained, on average, PRRS-free for two years. After eliminating PRRS, the average percentage of new outbreaks per year was 23%, similar to the national average of 20–25% (regardless of the previous PRRS status). Additional factors might contribute to the decision to pursue elimination, and further studies are warranted.

**Abstract:**

Background: Hesitation on eliminating Porcine Reproductive and Respiratory Syndrome virus (PRRSV) from breeding herds exists since it is difficult to predict how long the herd will remain virus-free. We aimed to estimate the time that breeding herds remained virus-free (naïve) after PRRSV elimination was achieved. Methods: Production systems voluntarily shared their breeding herds’ health status weekly between July 2009 and October 2021. PRRSV incidence rate and the total number of days a breeding herd remained virus-free were estimated. Results: A total of 221 (17%) herds reached the naïve status 273 times. The median time sites remained in this status was approximately two years. The overall PRRS incidence rate after sites achieved a naïve status was 23.43 PRRS outbreaks per 100 farm years. Conclusion: Estimates obtained here provide insights on how frequently and for how long sites remain naïve, which contribute to informing management practices for PRRS control.

## 1. Introduction

Porcine reproductive and respiratory syndrome (PRRS) is one of the most important swine infectious diseases due to its high economic burden [1,2]. It was first identified in the United States in the early 1990′s [3] and has remained endemic, with 20–40% of the breeding herds reporting an outbreak every year [4].

Several methods for herd level control (e.g., McRebel, gilt isolation and acclimation, vaccination) and elimination (e.g., whole herd depopulation and repopulation, herd closure and rollover) have been proposed [5,6]. Producers and veterinarians depending on the region, choose whether the herd is a candidate for the elimination of the wild-type or vaccine virus, known as stability, or elimination and rollover, indicating a seronegative and virus-free breeding herd, also known as negative or naïve [7,8]. Still, after successful control and elimination, some breeding herds will still be at risk for PRRS reintroduction due to regional disease pressure. Therefore, producers might be discouraged from pursuing a stable or naïve PRRS status, particularly in high-density regions. Instead, they might opt for enduring at an endemic or a stable status (seropositive animals) indefinitely through the vaccination or live-virus inoculation of recently introduced animals to maintain some immunity in the herd. However, although PRRS modified-live vaccines have been proven to mitigate the economic impact of an outbreak, it does not confer sterilizing immunity to heterologous infections [9,10,11], and if strict biosecurity practices are not in place to prevent new viral introductions, herds remain susceptible to infections.

In efforts toward system-wide, regional, or national PRRS elimination, a common hesitation for producers to implement an elimination strategy is the uncertainty of how long the herd will remain free of the virus. Here, we aimed to assess for how long farms that chose to eliminate PRRS and rollover their herds remained at a naïve status to ultimately help in informing decisions toward PRRS management.

## 2. Materials and Methods

Data for this study originated from the Morrison Swine Health Monitoring Project (MSHMP), which aims at developing tools for foreign animal disease preparedness while monitoring swine diseases such as PRRS in approximately 50% of the U.S. sow population [12]. Participating pig production systems voluntarily shared their breeding herd health status weekly based on their routine veterinary care and disease surveillance. For PRRS, statuses are classified as positive unstable, positive stable, provisional negative, or negative/naïve [8]. Positive unstable (status 1-S1) comprise seropositive shedding status and can be divided into high or low virus prevalence in the weanling piglet population. Positive stability (status 2-S2) is achieved when animals are seropositive but with no evidence of virus-positive weanling piglets after 90 days. In the provisional negative status (status 3-S3), naïve replacement animals are introduced and remain ELISA negative 60 days after introduction. Finally, the negative/naïve (status 4-S4) is when all previously infected animals have been removed from the herd. Additionally, S2 sites are sub-classified as 2fvi (S2fvi) if there was ongoing field virus exposure in gilts or 2vx (S2vx) if there was ongoing exposure with a live vaccine to gilts and/or sows.

The MSHMP’s weekly breeding herd PRRS health status data from July 2009 up to October 2021 was used. For this period, 1316 breeding herds from 40 pig production systems were included. From the enrolled herds, location data was available for 1247 herds, of which 52.8% (*n* = 658) were from the Midwest, 38.4% (*n* = 479) from the South, 6.4% (*n* = 80) from the Northeast, and 2.4% (*n* = 30) were from the West according to the U.S. Census [13]. The median sow herd size of these herds was 2500 (inter-quartile range –IQR: 1400–3900). No biosecurity or management information is requested under the MSHMP, but the air filtration status of each breeding site has been recorded over time. The total number of days between achieving S4 and the next change in status was calculated. Descriptive statistics of sites that reached S4 were calculated, and those that remained in S4 at the end of the study period were censored on 27 October 2021. Since sites that reached S4 closer to the end of the study period would artificially have a shorter naïve time, a survival analysis was conducted to assess the probability of experiencing a new PRRS introduction event (i.e., a change from S4 to S1) over time while at risk. Time at risk was defined as the time since the site reached S4 until the time of the new introduction event (failure) or the end of the study period (censoring). A Kaplan–Meier curve was constructed according to the year in which S4 was achieved. Additionally, the PRRS incidence rate was computed considering the number of sites that changed from S4 to S1 and the total contribution time in farm years (number of years between the initial change to S4 and the next change in status). A multilevel Poisson regression was used with the production system as a random effect variable and time at risk as exposure to estimate the overall incidence rate in this population. We constructed bivariate multilevel mixed-effects parametric survival models with site nested within the system as a random effect variable to account for multiple times achieving S4 and having PRRS reintroduced in the herd, as well as to account for the interdependencies of sites within the same production systems. These models were used to assess the effect of the year in which S4 was achieved, the region (Midwest, North East, South, and West) and air filtration status on the time to outcome (i.e., PRRSV occurrence) among sites that reached S4 during the studied period. Statistical analyses were made using STATA 15 [14].

## 3. Results

The follow-up time of the included breeding herds ranged between 1 and 12 years. During this period, 221 herds achieved S4 273 times (48 from S1, 167 from S2, 30 from S2fvi, 20 from S2vx, and 8 from S3). The number of sites reaching S4 each year ranged from 3 (2009) to 35 (2014), with an average of 21 herds reaching S4 each year throughout the studied period. Most sites that achieved S4 were from the Midwest (67.40%, *n* = 184), followed by the South (19.41%, *n* = 53), Northeast (6.96%, *n* = 19), and West (1.10%, *n* = 3), while 14 had no information on location. Filtration status was available for 139 of the sites that reached S4 status, of which 38, 5, and 18 were not filtered; partially filtered during the fall, winter, and spring seasons; or filtered year-round, respectively.

**Figure 1:**
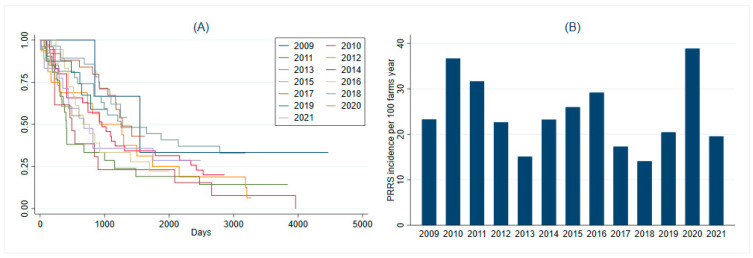
Kaplan–Meier curve on the probability of not having a PRRS outbreak according to the year the naïve status was achieved (**A**) and the PRRS incidence rate per 100 farm years by year in which sites became naïve (**B**).

The median number of days sites remained in S4 was 728 (IQR 280–1281) days, ranging between 7 and 3962 before changing to a different status. Three breeding herds remained in S4 for a period of 7 days. Among those, two switched to an S1 due to a wild-type virus introduction and one to status S2vx. The probability of an S4 farm remaining as such and not having a PRRS outbreak according to the year when S4 was achieved is shown through the Kaplan–Meier curve (Figure 1A).

In 91 out of the 273 times herds achieved the S4 classification, the status was successfully maintained until the end of the study period, whereas in 163 (59.71%) cases, herds eventually lost such status due to wild-type virus introduction. On 15 occasions, herds switched from S4 to S2vx as a result of herds being vaccinated with a modified-live vaccine. Information on the new status was not available for four S4 events that had ended during the study period. For sites that moved from S4 to S1, the median time between achieving status 4 to the new introduction was 602 days (inter-quartile range: 259–1050).

The PRRS incidence rate after sites reached a naïve status ranged from 10.65 to 38.83 outbreaks per 100 farm years, depending on the year the site reached S4 (Figure 1B). The overall incidence rate for the entire period was 22.52 PRRS outbreaks per 100 farm years. Adjusting for the production system, the overall incidence rate was 23.43 PRRS outbreaks per 100 farm years. Of all the 163 PRRS outbreaks following recorded naïve status, 34.97% (*n* = 57) occurred in the winter, whereas 27.61% (*n* = 45) occurred in the fall, 23.93% (*n* = 39) occurred in the spring, and 13.50% (*n* = 22) in the summer.

Bivariate survival models adjusted for multiple events in the same site and for the production system showed that air filtration and the year in which the site reached a naïve status did not differ in terms of time to PRRS introduction (Table 1). The year was used as a continuous variable, meaning the hazard ratio corresponds to a one-year increase. Regarding region, S4 sites in the Northeast and South were shown to have 25% and 31% of the hazard rate for a PRRS introduction than the Midwest S4 sites.

## 4. Discussion

S4 was achieved by 17% of the breeding herds during the study period. Those that reached S4 remained as such for a median of two years (or 738 days). However, it is important to note that events were censored at the end of the study period. Censoring might have particularly affected those that reached S4 more recently, possibly underestimating this average since about a third of status four events were still ongoing. Overall, for the correspondent of 100 S4 farms followed for one year, on average, 23 of them would have experienced a PRRS outbreak throughout this period. Although this is similar to the overall cumulative annual PRRS incidence observed in U.S. breeding herds through recent years, ranging approximately 20–25% [4], S4 farms, which are a subset of the overall cumulative incidence population, have historically been among the lowest PRRS incidence rate categories [4]. While this study does not address risk factors for PRRS outbreaks (rather factors associated with the time of a PRRS introduction), we found that most PRRS naïve herds that reported an outbreak occurred during the winter and fall seasons. This is consistent with the typical overall PRRS seasonality reported, even though regional variability regarding PRRS seasonality is also expected to be present [15,16].

Although the PRRS incidence rate fluctuated by the year in which sites reached S4, we found that the year reaching S4 was not statistically significant for the PRRS rate of introduction when accounting for sites and systems. This might be explained by the application of standardized PRRS management strategies consistently every year or by a lack of power in the study since, on average, only 21 sites reached a naïve status each year, ranging from three to 35. Additionally, the monitored population was not consistent throughout the years, as there was an open enrollment of production systems to the monitoring project during the study period. The yearly number of participating systems ranged from 19 to 40.

While the South and the Midwest are within the highest swine-dense regions in the U.S. with a similar number of sites participating in the MSHMP, the Midwest has an over three times higher number of sites reaching a naïve status than the South. We found that sites in the Northeast and South that reached S4 had a lower risk of PRRS introduction over time compared to sites that reached S4 in the Midwest. A possible explanation is that while over 20 systems participate in this monitoring project in the Midwest, the South and the Northeast are mostly dominated by less than five production systems. While the production system was accounted for in our model, the inherent heterogeneity of other factors such as PRRS prevention, control, and elimination philosophy affecting transmission and heterogeneity of production systems distribution might not have been captured. Moreover, previous studies showed that pig-dense areas (as is the case for most Midwest and Southern participants) were associated with higher PRRS incidence [16,17]. The PRRS risk also varied regionally according to season [15,16], which might also influence the system’s willingness to pursue elimination according to their perceived risk.

No difference in time to a PRRS introduction according to air filtration status was found in this study, even though lower PRRS incidence rates were reported in sites with year-round air filtration [16]. However, time to PRRS introduction was only assessed in farms that had reached a naïve status, which represents only 17% of all monitored herds. Sites that had opted to pursue a naïve status are likely different from those that opt to endure at positive stable statuses in terms of general biosecurity practices. That, together with the different regional distribution of sites that achieve S4 compared to sites that never achieve S4 and the perceived risk of investing in air filtration, likely explain this difference. In fact, all of the sites that achieved S4 and were either partially or year-round filtered were from the Midwest.

Other important potential benefits of maintaining S4 were not assessed in this study, such as the impact on their breeding and growing herd production performance since they are not facing the same health challenges as sites that do not seek elimination. This can be of particular importance and needs to be further investigated since a frequent alternative to elimination is the continuous exposure of gilts entering the herd to the modified-live vaccine (S2vx), which can lead to viremia, viral shedding and potential crossing of the placental barrier in pregnant sows [9,18,19]. Although studies have demonstrated the effects of vaccination compared to other control strategies on production parameters in the face of an outbreak [20], there is still a knowledge gap regarding the economic benefits of maintaining a status 2vx indefinitely instead of pursuing elimination.

## 5. Conclusions

Although we were unable to study other factors, such as the decision process to continue pursuing S4 and additional biosecurity practices implemented, among others that might impact days as S4 and the PRRS incidence rate, our study provides insights on how frequently sites are striving to eliminate and how long can they maintain their naïve status.

## Figures and Tables

**Table 1:** Bivariate multilevel mixed-effects parametric survival models with sites nested within the production system as a random effect showing hazard ratio (HR) of PRRS outbreaks in sites that reached a naïve status from July 2009 to October 2021.

Characteristic	N Sites	N PRRS Breaks	HR	95%CI	*p*-Value
Air filtration					
Not filtered	63	25	1	-	-
Partially filtered	12	7	1.06	0.32–3.53	0.92
Year-round	64	18	1.59	0.62–4.08	0.33
Year naïve			0.98	0.91–1.06	0.58
Region					
Midwest	184	123	1	-	-
Northeast	19	4	0.25	0.07–0.88	0.03
South	53	24	0.31	0.14–0.68	0.004
West	14	10	1.15	0.22–5.97	0.87

## Data Availability

The dataset used is privately owned by the production systems. Data are, however, available from the authors upon reasonable request from the corresponding author and with permission of the production systems involved, but restrictions might apply.
